# Cardiovascular magnetic resonance guided ablation and intra-procedural visualization of evolving radiofrequency lesions in the left ventricle

**DOI:** 10.1186/s12968-018-0437-z

**Published:** 2018-03-15

**Authors:** Philippa R. P. Krahn, Sheldon M. Singh, Venkat Ramanan, Labonny Biswas, Nicolas Yak, Kevan J. T. Anderson, Jennifer Barry, Mihaela Pop, Graham A. Wright

**Affiliations:** 1 Department of Medical Biophysics, University of Toronto, Toronto, ON Canada; 2 Sunnybrook Research Institute, Toronto, ON Canada; 3 Schulich Heart Research Program, Sunnybrook Research Institute, Toronto, ON Canada; 4 Division of Cardiology, Schulich Heart Centre, Sunnybrook Health Sciences Centre, Toronto, ON Canada; 5 Faculty of Medicine, University of Toronto, Toronto, ON Canada

**Keywords:** Image-guided intervention, Catheter ablation, Tissue characterization, Arrhythmias

## Abstract

**Background:**

Radiofrequency (RF) ablation has become a mainstay of treatment for ventricular tachycardia, yet adequate lesion formation remains challenging. This study aims to comprehensively describe the composition and evolution of acute left ventricular (LV) lesions using native-contrast cardiovascular magnetic resonance (CMR) during CMR-guided ablation procedures.

**Methods:**

RF ablation was performed using an actively-tracked CMR-enabled catheter guided into the LV of 12 healthy swine to create 14 RF ablation lesions. T_2_ maps were acquired immediately post-ablation to visualize myocardial edema at the ablation sites and T_1_-weighted inversion recovery prepared balanced steady-state free precession (IR-SSFP) imaging was used to visualize the lesions. These sequences were repeated concurrently to assess the physiological response following ablation for up to approximately 3 h. Multi-contrast late enhancement (MCLE) imaging was performed to confirm the final pattern of ablation, which was then validated using gross pathology and histology.

**Results:**

Edema at the ablation site was detected in T_2_ maps acquired as early as 3 min post-ablation. Acute T_2_-derived edematous regions consistently encompassed the T_1_-derived lesions, and expanded significantly throughout the 3-h period post-ablation to 1.7 ± 0.2 times their baseline volumes (mean ± SE, estimated using a linear mixed model determined from *n* = 13 lesions). T_1_-derived lesions remained approximately stable in volume throughout the same time frame, decreasing to 0.9 ± 0.1 times the baseline volume (mean ± SE, estimated using a linear mixed model, *n* = 9 lesions).

**Conclusions:**

Combining native T_1_- and T_2_-based imaging showed that distinctive regions of ablation injury are reflected by these contrast mechanisms, and these regions evolve separately throughout the time period of an intervention. An integrated description of the T_1_-derived lesion and T_2_-derived edema provides a detailed picture of acute lesion composition that would be most clinically useful during an ablation case.

## Background

Ventricular tachycardia (VT) ablation is now frequently performed, with the rate of use of these procedures increasing substantially in the last decade [1]. Recurrence of VT after a single ablation procedure remains high, with 35% of patients who receive initially successful ablation treatment later experiencing VT recurrence during 6–23 month follow-up periods [2]. VT recurrence after an ablation procedure is complex and may be related to the inability to localize the critical circuit, or, when the circuit is defined, inability to obliterate the critical channels [3]. Once the critical isthmus of a VT circuit is isolated, radiofrequency (RF) ablation is performed with the hope of creating a necrotic lesion (permanent injury) at the putative isthmus, rendering VT non-inducible. The insult of ablation also leads to edema (reversible injury) surrounding the ablation site [4–8]. This reversible injury is thought to result in transient conduction block, with conduction recovering once the edema has resorbed [9–11], potentially leading to late arrhythmia recurrence. The ability to detect whether the arrhythmogenic substrate has been permanently destroyed, as evidenced by the presence of a lesion at a critical ablation site, may be invaluable and provide an additional intra-procedural endpoint to gauge long-term procedural success.

Cardiovascular magnetic resonance (CMR)-based identification of the critical isthmus sites may be used to guide ablation toward these targets [12], and the therapeutic lesions themselves can be directly visualized using native (non-contrast enhanced) CMR [4–8, 10, 13–16].

Ablation lesions visualized using native T_1_ reflect injury that is associated with lethal heating [17], that persists at least for 3 weeks [13], and that correlates to chronic lesions [6], therefore likely represents permanent injury. Conversely, myocardial edema is visualized with native T_2_ [18] and is transient, largely resolved within 3 months as seen in follow-up T_2_-weighted imaging after atrial ablation [8]. The T_1_-derived lesion and T_2_-derived edema are individually significant in clinical ablations as the distributions of these injured regions could determine whether electrical block will remain permanently or resolve after healing (leading to recurrent VT). To evaluate these regions, this study includes concurrent native T_1_- and T_2_-based imaging to compare their relative extents and to construct a comprehensive understanding of the acute lesion composition.

A major potential value of intra-procedural CMR imaging lies in the ability to directly visualize and interpret the functional effect of therapeutic ablation lesions during the period when intra-procedural modification is possible. It is therefore important to identify how different aspects of CMR information evolve in this time window and how this information might be related to conventional electrophysiology (EP) endpoints. This study aims to characterize the evolution of lesions, as previously suggested to take place [16], but using concurrent imaging of both the T_2_-derived edema and T_1_-derived lesion to provide volumetric measurements of each. We hypothesize that the acute ablation-induced T_2_-derived edema evolves during the acute time frame while the T_1_-derived lesion remains at a consistent size. Furthermore, adding T_2_ mapping to this imaging framework provides unambiguous quantification of edema severity. These techniques to study the acute lesion composition build upon prior work studying lesion characteristics via the surrogate measure of gadolinium kinetics [19]. This study aims to show temporal characteristics of the native T_1_-derived lesion and T_2_-derived edema within a time frame of particular relevance to direct CMR guidance of ablative therapies, extending the current understanding of lesion evolution previously established using native-contrast CMR [5] to an earlier time frame. Insights from lesion evolution could be applied to both CMR-guided and traditional ablation cases.

## Methods

### Animal preparation

CMR-guided RF ablation was performed in vivo in 12 healthy Yorkshire swine (58 ± 18 kg). The Animal Care Committee of Sunnybrook Research Institute approved all protocols. All animals received an intramuscular injection of ketamine (33 mg/kg) and atropine (0.05 mg/kg) pre-anaesthesia, followed by isoflurane gas (1–5%) continuously delivered via mechanical ventilation to maintain the surgical stage of anaesthesia. A sheath in one carotid artery acted as a port for catheter introduction. To mitigate arrhythmia, a bolus of amiodarone (75 mg) was given prior to the intervention, in addition to lidocaine (20 mg) as needed.

### CMR-guided intervention

**Fig. 1:**
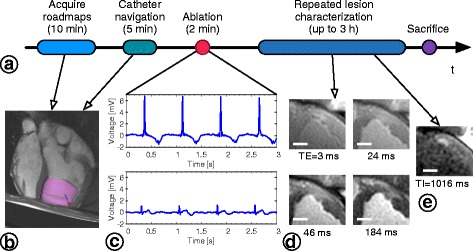
CMR-guided intervention experimental workflow. **a** Data acquisition and repeated lesion imaging performed throughout CMR-guided interventions. **b** Still frame of the actively-tracked catheter tip (blue and red arrow) during navigation overlaid on anatomical roadmap images in VURTIGO [22]. The LV endocardial surface (pink shell) was automatically delineated [41] and displayed to assist navigation. **c** Bipolar intracardiac electrogram (EGM) traces recorded from the catheter held at the ablation site immediately before ablation (upper panel) and approximately 3 s after stopping RF delivery (lower panel). **d** Anterior wall RF lesion visualized in T_2_-weighted images. Each stack of T_2_-weighted images was acquired at 4 TEs to construct T_2_ maps (16 s breath-holds, approximately 4 ± 2 min in duration), which were reconstructed offline, along with all image analysis. **e** RF lesion visualized in T_1_-weighted IR-SSFP. IR-SSFP images were acquired in a stack of several 2D images with each slice requiring 1 breath-hold 16 s long. Scale bars = 1 cm

The entire intervention was performed within a 1.5 T wide-bore scanner (MR450w, General Electric Healthcare Waukesha, Wisconsin, USA). Figure 1 illustrates the interventional workflow and timing of data collection throughout. A 4-channel anterior cardiac array coil was used for all imaging, and was connected to the scanner bed separately from the catheter tip tracking coils. The CMR-enabled EP hardware configuration has been described previously [20].

Pre-procedure scans included short- and long-axis stacks of 2D balanced steady-state free-precession (bSSFP; CINE) images to serve as anatomical roadmaps (20 cardiac phases across 1 R-R interval, TR/TE = 5.2/1.9 ms, resolution = 1.25 × 1.5 mm, slice thickness = 6 mm). Both orientations were acquired at a rate of 1 breath-hold (approximately 14 s) per slice over 8 ± 3 min in total.

A 9 F CMR-enabled catheter (Imricor Medical Systems, Burnsville, Minnesota, USA) equipped with 2 micro-receive coils and 2 electrodes (3.7-mm tip electrode and 3.5-mm inter-electrode spacing) was used for the entire intervention. Active catheter tracking using a projection sequence [21] (FOV = 60 cm, flip angle = 5°, TR = 14.3 ms, tracking rate = 23 fps) was implemented in RTHawk software (HeartVista Inc., Menlo Park, California, USA). The catheter tip position was updated in real time and visualized overlaid on static anatomical roadmap images (Fig. 1b) simultaneously in VURTIGO, 4D visualization software for cardiovascular interventional guidance (Sunnybrook Research Institute, Toronto, Ontario, Canada) [22]. Bipolar intracardiac electrogram (EGM; Fig. 1c) traces from the catheter tip’s electrodes were recorded by the Advantage-MR system (Imricor Medical Systems). EP traces and VURTIGO catheter navigation visualization were displayed on monitors in the CMR control room and on CMR-compatible monitors near the scanner bed to assist the interventionalist. Once the catheter was inserted into the heart to access the LV via the aortic valve, ablations were delivered from the catheter’s distal electrode (1500 T14 generator, St Jude Medical, St. Paul, Minnesota, USA) with 17 mL/min irrigation and with the dispersive electrode placed on the animal’s back. Ablations were delivered endocardially, in accordance with the more common clinical ablation approach (details given in Table 1). Conservative ablation parameters were chosen to avoid inducing arrhythmia during ablation. Ablation was generally performed in similar locations for consistency, but targeting accuracy was not directly evaluated as no specific targeting was used for lesion placement. Vital signs including electrocardiogram (ECG), end-tidal CO_2_, and peripheral capillary oxygen saturation were monitored using the Expression CMR Patient Monitor (Invivo Corp., Gainesville, Florida, USA). Peripheral pulse sensing from each animal’s foot was sufficient for robust cardiac gating, and respiratory motion was frozen during imaging by pausing mechanical ventilation.

**Table 1:** Summary of ablation details and imaging data

Lesion #	Location	RF power / Duration	T_2_ map time span [min]	IR-SSFP time span [min]	MCLE	Gross pathology lesion diameter [mm]	Subject mode of death
1	Anterolateral wall	30 W/60 s	40–60	*	+	8.0	Arrhythmia
2	Anteroseptal wall	30 W/60 s	19–56	*	+	6.8	Euthanasia
3	Anterior wall	30 W/60 s	13–73	24–81	+	10.1	Euthanasia
4	Anterolateral wall	30 W/60 s	8–68	*	+	10.3	Euthanasia
5	Anterior wall	30 W/90 s	Not used due to artifact	33–55	–	11.8	Arrhythmia
6	Anteroseptal wall, apical	30 W/90 s	10–224	36–246	+	8.1	Arrhythmia
7	Anterolateral wall, apical	30 W/70 s	8–64	*	+	9.1	Arrhythmia
8	Anterior wall	30 W/100 s	3–172	20–184	–	11.9	Arrhythmia
9	Anterior wall	30 W/120 s	11–71	*	–	4.3	Arrhythmia
10	Anterior wall	35 W/120 s	11–127	18–132	–	7.9	Arrhythmia
11	Anterior wall	35 W/120 s	6–109	23–114	+	8.7	Euthanasia
12	Anterior wall	35 W/120 s	6–98	15–107	+	12.1	Arrhythmia
13	Anterior wall	30 W/90 s	9–171	19–184	+	7.1	Euthanasia
14	Anteroseptal wall	35 W/120 s	21–250	13–245	+	7.4	Euthanasia

Abbreviations: *IR-SSFP* inversion recovery balanced steady-state free precession, *MCLE* multi-contrast late enhancement, *RF* radiofrequency

T_2_ map and IR-SSFP time spans indicate the first and last acquisitions of each type (* = lesion not clearly apparent, likely due to partial-volume effects, +/− = acquired/not acquired). Lesions 3 and 4 were created in the same individual, likewise for lesions 6 and 7

### CMR imaging protocol

Imaging began immediately after catheter withdrawal (as early as 3 min after the start of ablation) to visualize the lesion near the catheter contact point. The two primary native contrast (non-contrast enhanced) imaging sequences used were alternated regularly at consistent slice locations to evaluate RF lesion temporal evolution.

T_2_ mapping was performed using a previously validated T_2_-prepared spiral sequence to detect regional T_2_ changes near the ablation site reflecting inflamed edematous tissue (4 TEs between 3 and 184 ms, TR = 2 R-R intervals, 10 interleaves, 3072 points, FOV = 240 mm, readout bandwidth = 125 kHz, effective resolution = 1.3 × 1.3 mm, slice thickness = 6 mm). Images at one TE were acquired per breath-hold (approximately 16 s), and complete maps were acquired in 4 ± 2 min.

An IR-SSFP sequence generated images with various inversion times at 40 cardiac phases across 2 R-R intervals [23]. Typical acquisition parameters were: views/segment = 16, flip angle = 45°, TR/TE = 5.6/2.0 ms, readout bandwidth = 62.5 kHz, FOV = 240 mm, matrix = 192 × 160, and slice thickness = 6 mm. Although SSFP sequences yield best quality with shorter TRs (e.g., TR = 2.7–4 ms at 1.5 T [7, 23]), this TR was longer than ideal in this wide-bore system with lower gradient specifications. This was the shortest achievable TR while maintaining other sequence parameters. Local shimming was performed to mitigate any off-resonance effects, and dark-band artifacts were not observed in the regions of interest.

RF ablation lesions were hyper-enhanced in IR-SSFP images due to shorter T_1_ compared to the healthy myocardium [7, 24]. TIs longer than approximately 700 ms (yielding optimal contrast for lesion core visualization [7, 13, 25]) were set to occur within diastole. Image acquisition was performed at 1 slice per breath-hold (each approximately 16 s), such that 5 slices with 2 rest breaths between each slice could be acquired within 2 min. Sequence parameters were adjusted slightly to accommodate animal size and heart rate (typically 75–95 bpm).

Native-contrast imaging was repeated up to approximately 3 h post-ablation. At the end of the CMR study a bolus of Gd-DTPA (0.2 mmol/kg, Magnevist, Bayer Healthcare Pharmaceuticals, Berlin, Germany) was injected for contrast-enhanced imaging to confirm the pattern of ablation. The IR-SSFP sequence was repeated after contrast agent injection (referred to as MCLE [23]), acquired at 1 slice per breath-hold, at 6 ± 5 min post-injection. Four of 12 animals succumbed to arrhythmia before the end of the imaging study when MCLE images were to be acquired. Animals were not moved during the CMR study; therefore, all images were well aligned post-ablation (confirmed via anatomical landmarks in corresponding images).

### *Ex vivo* examination

After the imaging study (within hours of ablation), animals were euthanized and the hearts explanted immediately, then preserved in a 10% formalin solution. Hearts were embedded in dental alginate gel and sliced to 4-mm blocks aligned to the imaging plane for gross examination and measurement of lesion dimensions. Tissue blocks were sliced to 4-μm sections and stained with Hematoxylin and Eosin (H&E) and Masson’s trichrome (MT) to emphasize tissue morphological changes and viability, respectively. Slides were scanned under light microscopy (Leica SCN400 F, Leica Microsystems, Wetzlar, Germany) at 20× magnification (0.5 μm resolution) to observe features of healthy tissue, the lesion, and peripheral regions around the ablation site. Edema extent was not assessed *ex vivo* given the challenge of determining associated borders in tissue sections. CMR images were taken as a more accurate representation of the extent of edema in vivo. Observations from histopathology were qualitative in support of the image-based observations of ablation injury patterns.

### Data analysis & statistics

All analysis was performed offline in MATLAB (MathWorks, Inc., Natick, Massachusetts, USA). Images were interpolated to a 256 × 256 matrix (DICOM format) before processing. T_2_ maps were generated using a previously-validated 3-parameter model [26] (approximately 15 s per map using non-optimized code). Regions of edematous tissue exhibiting increased T_2_ were segmented semi-automatically by applying a threshold of 3 standard deviations (SDs) above mean T_2_ in healthy tissue (similarly to the approach used in a prior ablation study [27]). Morphological operations were applied to this mask to remove noise and other erroneous pixels (MATLAB Image Processing Toolbox), identify edema as contiguous regions of segmented pixels, and fill these regions while preserving borders. This semi-automatic approach was implemented to yield reproducible volumes despite the often-diffuse quality of edema, and could be performed within approximately 2 min per slice.

IR-SSFP images acquired at TIs longer than approximately 700 ms were selected for lesion segmentation, with matching contrast in subsequent acquisitions. The T_1_-derived lesion volumes were segmented semi-automatically using a threshold of 2 SD above adjacent healthy myocardial signal intensity (SI), and correlated well with an expert viewer’s manual delineation of lesion volumes (Pearson’s *r* = 0.95, intercept = − 0.078, slope = 1.2, *p* < < 0.0001; 2 SD segmented volumes larger by 0.0017 ± 0.10 mL overall; 95% limits of agreement [− 0.030, 0.033]). Segmentation required approximately 3 min per slice.

All native T_1_-weighted images and T_2_ maps included in analysis were acquired in vivo. To assess temporal changes in the T_1_-derived lesions and T_2_-derived edema, CMR-based volume measurements were normalized to those from the initial imaging time point post-ablation. We report all post-ablation times with respect to the start of ablation, as lesion formation commences at this point. Lesion volume temporal evolution was evaluated using a linear mixed model (LMM) to account for clustering by animal and baseline differences (MATLAB Statistics Toolbox). The ratio of T_2_-derived edema and T_1_-derived lesion volumes was calculated to provide a direct comparison of these volumes across individual ablations at different time intervals, assessed using analysis of variance (ANOVA). T_2_ evolution was assessed in three key ROIs: healthy tissue; T_1_-derived lesions; and edematous regions (the largest edematous ROIs for each ablation, to examine changes in a consistent tissue region). LV wall thickness and lesion transmurality (lesion depth divided by wall thickness) were also compared at different time points.

Lesion diameters measured manually from the final in vivo IR-SSFP images acquired were compared to those from morphologically matched gross tissue slices (the gold standard for lesion formation). Lesions in MCLE images were segmented manually to compare lesion volumes derived from native-contrast and contrast-enhanced images. Both comparisons were performed using Bland-Altman analysis. All measurements are reported as the mean ± SD unless indicated otherwise, and *p* < 0.05 was considered statistically significant.

## Results

### RF lesion temporal evolution

**Fig. 2:**
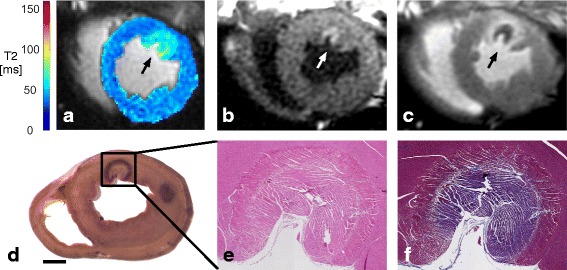
RF lesion visualization using native-contrast and contrast-enhanced CMR, gross pathology, and histopathology. **a** T_2_ map (74 min post-ablation) demonstrating T_2_ elevation associated with edema near the ablation site (arrow). **b** IR-SSFP (TI = 730 ms, 81 min post-ablation) demonstrating the hyper-enhanced lesion. **c** MCLE (TI = 805 ms, 106 min post-ablation, approximately 6 min post-Gd injection), demonstrating the dark region of microvascular obstruction, at the lesion centre, with bright surrounding tissue. **d** Gross pathology (with a second lesion slightly out of plane; scale bar = 1 cm). Magnified (**e**) H&E and (**f**) MT stained lesion tissue sections

**Fig. 3:**
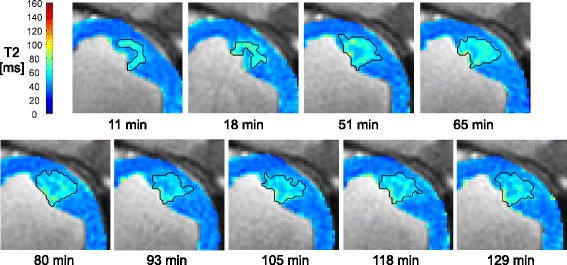
T_2_-derived edema development with time post-ablation. Anterior LV wall RF lesion visualization in T_2_ maps acquired 11–129 min post-ablation. Black lines delineate segmented T_2_-derived edematous regions

The native T_1_-derived lesion and T_2_-derived edema were clearly visualized and reflected the characteristic teardrop shape of RF lesions observed in corresponding contrast-enhanced images, gross pathology, and histopathology (Fig. 2). Elevated T_2_ indicating edema surrounding the ablation site was evident in the first T_2_ maps acquired as early as 3 min after the start of ablation. T_2_ maps were analyzed across 13 ablations visualized in 1–3 adjacent imaging slices in 11 animals. Each T_2_-derived edematous region was visualized at 5 ± 3 (median 4) time points following ablation. These regions tended to initially appear more focal and localized to the lesion border, assuming a more diffuse appearance by later time points (Fig. 3).

**Fig. 4:**
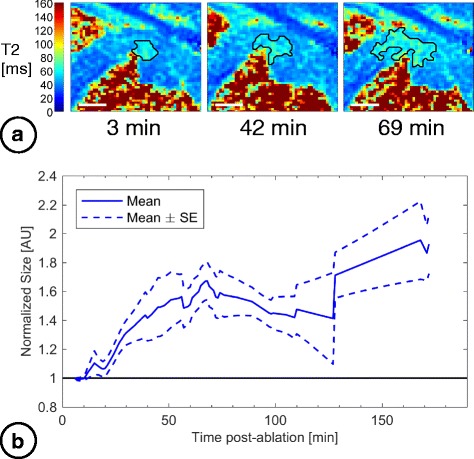
Evolution in volumes of acute T_2_-derived edema post-ablation. **a** Anterior wall RF lesion visualized in T_2_ maps with T_2_-derived edema delineated (black lines; scale bars = 1 cm). **b** Cumulative volume of T_2_-derived edema normalized to baseline (the first image acquisition), with time after ablation. Interpolated data from 13 lesions were used to calculate mean and SE curves. All acquired data were used; the mean curve is shown only out to 172 min due to insufficient data beyond this point to create error bars

The volume of T_2_-derived edema was 0.77 ± 0.55 mL (*n* = 13) at the baseline measurements from the earliest T_2_ maps acquired (median 10 min post-ablation). The development of edema is shown in Fig. 4, illustrating the overall increase in volume compared to baseline. Normalized edema volume increased significantly beyond baseline (LMM slope = 0.003 ± 0.001, mean ± SE, *p* = 0.009, intercept = 1.20 ± 0.01, *p* < 0.0001). Using this model, T_2_-derived edema expanded to an estimated 1.7 ± 0.2 (mean ± SE) times the baseline volume by 180 min post-ablation. Although the overall trend indicated increasing volume, in 6/13 of T_2_-derived edematous regions the volume increased then later dropped (while still remaining 1.3 ± 0.6 times larger than baseline). The maximum T_2_-derived edema volume estimated across all lesions was 1.5 ± 1.0 mL. T_2_-derived edema did not consistently develop concentrically around the catheter contact point.

**Fig. 5:**
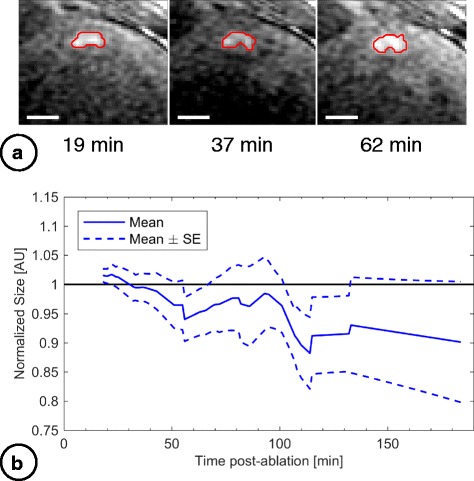
Monitoring volumes of acute T_1_-derived lesions post-ablation. **a** Anterior wall RF lesion visualized using IR-SSFP with lesion delineated (red lines; scale bars = 1 cm). **b** Cumulative volume of the T_1_-derived lesions normalized to baseline (the earliest imaging time) with time after ablation. Interpolated data across 9 lesions were used to calculate mean and SE curves. All acquired data were used; the mean curve is shown only out to 184 min due to insufficient data beyond this point to create error bars

The TIs used for T_1_-derived lesion visualization using IR-SSFP were TI = 831 ± 150 ms. These lesion volumes were measured in 9 lesions in 9 animals, each visualized in 1–3 adjacent imaging slices at 5 ± 2 (median 4) time points post-ablation (summarized in Fig. 5). The mean volume from the baseline IR-SSFP images was 0.48 ± 0.23 mL (*n* = 9, median 20 min post-ablation). Cumulatively, a small trend towards decreasing normalized T_1_-derived lesion volume was detected up to 180 min post-ablation (LMM slope = − 0.0006 ± 0.0003, mean ± SE, *p* = 0.09, intercept = 1.01 ± 0.04, *p* < 0.0001). Using this model, T_1_-derived lesion volumes reached an estimated 0.9 ± 0.1 (mean ± SE) times the baseline volume by 180 min post-ablation.

**Fig. 6:**
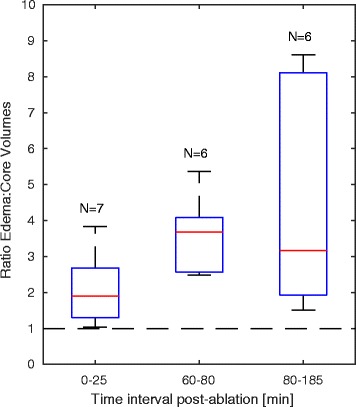
Ratio of T_2_-derived edema volumes to T_1_-derived lesion volumes post-ablation. T_2_-derived edema volume compared to T_1_-derived lesion volume at corresponding time points within 3 time intervals: initial lesion measurements within 0–25 min post-ablation; lesion progression within 60–80 min; and towards the end of CMR studies within 80–185 min post-ablation

The T_2_-derived edema consistently encompassed T_1_-derived lesions at individual ablation sites (Fig. 6), further supporting the distinctive tissue regions identified with each imaging contrast. The edema-lesion volume ratio was initially 2.1 ± 1.0 (0–25 min post-ablation, 95% CI [1.1, 3.0]). This ratio increased to 3.6 ± 1.1 (60–80 min; 95% CI [2.5, 4.8]), and reached 4.4 ± 3.2 (95% CI [1.1, 7.8]) by the final time interval at 80–185 min post-ablation. Although differences between intervals were not statistically significant (*p* = 0.2, ANOVA), volume ratios were greater than 1 within each interval (*p* < 0.05). The RF energy delivered did not correlate to the maximum measured T_1_-derived lesion or T_2_-derived edema volumes (*r* = − 0.2, 0.4; *p* = 0.7, 0.2, Spearman’s rho test); however, true energy deposition likely varied with catheter contact force, orientation, and blood flow.

**Fig. 7:**
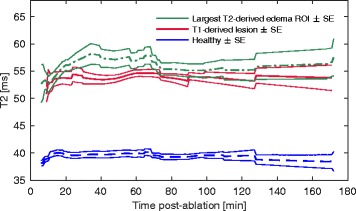
Longitudinal T_2_ post-ablation in healthy tissue, T_2_-derived edematous regions, and T_1_-derived lesions. Time course of: T_2_ in healthy tissue (*n* = 13 volumes); T_2_-derived edematous regions (*n* = 13); and T_1_-derived lesions (*n* = 8). Traces are shown for the time course corresponding to that shown in Figs. 4 and 5

T_2_ was evaluated in 3 critical regions: the T_1_-derived lesion; maximum T_2_-derived edematous region; and healthy tissue (Fig. 7). T_2_ within the T_1_-derived lesion ROIs increased between 0 and 25 min (53 ± 10 ms) and 60–80 min post-ablation (55 ± 8 ms, *p* = 0.04), then returned to the initial T_2_ at 80–185 min (53 ± 7 ms, *p* = 0.08). T_2_ within the largest edematous regions was significantly higher than in the T_1_-derived lesions overall (*p* < 0.001) and initially increased between 0 and 25 min (53 ± 15 ms) and 60–80 min post-ablation (58 ± 13 ms, *p* < 0.001), then decreased at 80–185 min, still remaining significantly higher than baseline (56 ± 15 ms, *p* < 0.001). T_2_ within both T_1_-derived lesions and largest edematous regions were significantly higher than in healthy tissue (39 ± 5 ms, *p* < 0.001), and T_2_ in healthy tissue did not change significantly during corresponding time intervals (*p* = 0.2, ANOVA).

The lesions were not generally transmural. T_2_-derived edema was 76 ± 18% transmural at baseline (15 ± 10 min post-ablation), reaching 79 ± 22% at final measurement (120 ± 67 min; *p* = 0.9). Similarly, transmurality of the T_1_-derived lesion did not change significantly from initial measurements of 52 ± 10% (21 ± 7 min post-ablation) to 50 ± 11% (162 ± 63 min; *p* = 0.3). LV wall swelling occurred at the ablation sites between pre-ablation (8 ± 2 mm) and baseline post-ablation measurements (11 ± 2 mm; *p* = 0.01), but further swelling through to the end of the studies was not substantial (12 ± 2 mm; *p* = 0.2). Wall thickness at remote sites remained consistent with pre-ablation measurements of 9 ± 2 mm, reaching 8 ± 2 mm initially post-ablation (*p* = 0.5) and 9 ± 2 mm (*p* = 0.9) at the end of the studies.

### Final pattern of ablation

MCLE validation imaging reflected the lesion geometry consistently observed in corresponding intra-procedural images (Fig. 2). The MCLE-derived lesion volumes were 0.75 ± 0.39 mL (*n* = 9), significantly larger than those measured in the corresponding final IR-SSFP acquisitions (*n* = 6; bias = 0.41 ± 0.31 mL, *p* = 0.03; 95% limits of agreement [0.08, 0.74]), and smaller than the corresponding final T_2_-derived edema volumes (*n* = 9; bias = 0.45 ± 0.79 mL, *p* = 0.2; 95% limits of agreement [− 0.16, 1.1]).

Lesion diameters observed in gross pathology slices and in the final IR-SSFP acquisition were well correlated (*n* = 9 lesions, Pearson’s *r* = 0.87, slope = 0.9, *p* = 0.003), with a relatively small bias of 0.4 ± 1.0 mm (gross lesion diameter = 9.7 ± 2.0 mm, T_1_-derived lesion diameter = 10.1 ± 2.0 mm; 95% limits of agreement [− 0.41, 1.2]).

### Histological characteristics of RF lesions

RF lesions exhibited the characteristic pale pink core of thermal injury bordered by a dark rim in gross pathology (Fig. 2d), also reflected in histological sections (Fig. 2e-f). Morphologic changes were emphasized using H&E (cytoplasm and extracellular matrix are stained pink, nuclei stained purple), and viability emphasized using MT (ischemic or necrotic tissue is stained purple, healthy viable myocytes red, and connective tissue blue).

**Fig. 8:**
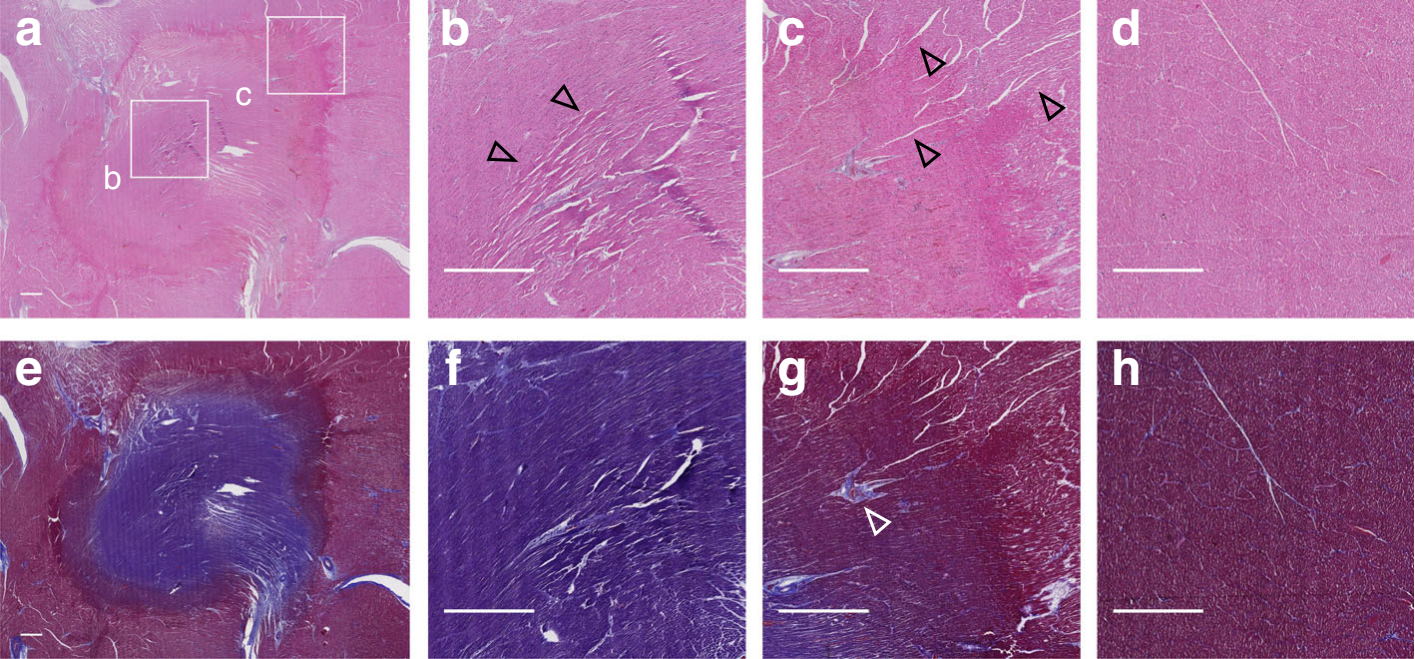
Histological sections of ablated tissue. Full extent of the lesion is shown in (**a**) stained with H&E, which highlights morphologic changes. **e** Full extent of the lesion stained with MT. **b** Magnified H&E section from the lesion core, showing cells exhibiting redistribution of extra/intracellular fluids due to thermal coagulation (resulting in the darker appearance of tissue). **c** Magnified H&E section from the lesion rim. Wide interstitium (black arrowheads) is evident relative to (**d**), healthy remote tissue. **f**-**h** Magnified MT sections from tissue zones corresponding to the H&E panels above. In the lesion rim, panels (**c**) and (**g**), extravasated red blood cells (RBCs) and vessels containing thrombosed RBCs (white arrowhead), blocking blood flow, are evident. All scale bars = 1 mm

The lesion core in H&E sections (Fig. 8a-b) exhibited disrupted cellular architecture consistent with thermal coagulation [28, 29] and the purple colour of the corresponding MT section (Fig. 8f) suggested non-viability of this tissue. The surrounding rim (Fig. 8c) was also distinguished by altered cellular architecture, and contained extravasated red blood cells, evidence of hemorrhage. The purple-to-red colour gradient outward from the lesion rim (Fig. 8g) suggested an increasing proportion of viable myocytes with distance from the catheter contact point. Interstitial space was increased conspicuously throughout the lesion (Fig. 8b-c) and extending beyond the lesion rim (compared to healthy tissue; Fig. 8d), consistent with observations of broad T_2_-derived edematous regions.

## Discussion

### Main findings

Native-contrast CMR was used to construct a comprehensive description of RF lesion composition and temporal evolution in the LV. Intra-procedural T_1_- and T_2_-based imaging performed concurrently each reflect a distinctive region of tissue injury, evolving separately throughout the time period of an intervention. Acute T_1_-derived lesions (thought to represent the permanent lesion) remained at a relatively stable size whereas the broader T_2_-derived edema (likely transient injury) was dynamic, tended to expand over time, and consistently extended beyond T_1_-derived lesions.

### Lesion visualization immediately post-ablation

By performing ablation under CMR guidance, intra-procedural visualization of the T_1_-derived lesions and T_2_-derived edema was achieved within minutes after ablation. T_1_ contrast after ablation is believed to originate from oxidation of ferrous (Fe^2+^) to ferric (Fe^3+^) iron associated with the transformation of hemoglobin to paramagnetic methemoglobin occurring at 55–65 °C [7, 30], and a recent study using real-time CMR thermometry during ablation showed that native T_1_ contrast reflects tissue which received a lethal thermal dose [17].

The acute lesions appeared desiccated in the central core, from T_2_ maps showing a zone of shorter T_2_ than the surrounding tissue (Fig. 3). Ablation-induced injury may be explained by comparison to general descriptions of thermal injury and edema pathogenesis [28, 31]. A sub-lethal thermal dose in tissue surrounding the lesion core is likely the cause of edema formation there. Microvessel permeability may be initially increased around the lesion core due to the release of biochemical factors such as histamine into injured tissue, leading to accumulation of fluid (edema).

### Acute lesion evolution minutes to hours post-ablation

Native-contrast CMR facilitated repeated measurement of both the T_1_-derived lesion and the T_2_-derived edema to assess their acute evolution throughout the interventional procedure. The evolution of native CMR contrast is of particular importance since lesion measurements can be repeated easily at different points in the procedure, while gadolinium-enhanced studies are limited to a single injection of contrast.

The acute T_1_-derived lesions appeared largely stable in volume, corroborating and extending existing studies that showed T_1_-derived lesions visualized acutely had a consistent extent which corresponded well to lesions visualized hours, weeks, and months post-ablation [5, 6, 13, 17]. By combining the results from this study with the existing data on T_1_-derived lesions, we suggest that the spatial extent of these lesions is established immediately after ablation, with damaged tissue later replaced by fibrotic scar. The subtle trend seen in this study toward decreasing T_1_-derived lesion size with time could be explained by passive edema diffusion toward the initially desiccated centre of the lesion, driven by reduced interstitial pressure there. Interstitial pressure changes in burned tissue are generally driven by the release of particles which drive osmotic pressure, drawing fluid into the interstitium [31]. Increasing water content would elevate local T_1_, opposing the reduction of T_1_ due to iron transformation.

The interstitium in thermally injured tissue tends to become more compliant, perpetuating fluid accumulation, with damage to matrix molecules such as collagen and hyaluronic acid. Increased permeability of injured microvessels leads to continued fluid leakage into the interstitial space [31]. Microvascular injury has previously been detected up to 6 mm beyond the pathological RF lesion core [29, 32], and the resulting ischemic effects likely contributed to the continuing increase in T_2_-derived edematous volumes and the increasing T_2_ within these regions up to approximately 60 min post-ablation (Fig. 7). The edema likely reached a stable extent once opposing interstitial and vascular pressures equalized, then later a more homogeneous fluid distribution with passive diffusion, consistent with the decreasing then stable T_2_ after the initial peak seen in edematous tissue towards the end of the CMR studies.

Non-concentric edema development of the T_2_-derived edema, and stable transmurality, could reflect preferential fluid spread occurring along cleavage planes between myocardial sheets, in line with LV muscle fibres that primarily run circumferentially. Beyond 120 min post-ablation, edematous regions appeared to have more diffuse borders (e.g., Fig. 3). Variable volume measurements at later time points could result from cases in which T_2_ elevation at the ablation site dropped below the 3 SD threshold; intensity-based segmentation is less robust to this diffuse pattern. Despite volume variability, the T_2_-derived edema volumes were still larger than immediate post-ablation dimensions (Fig. 4) and the corresponding T_1_-derived lesion volumes (Fig. 6).

Prior studies using T_2_-weighted CMR showed little change in edematous area beyond 3 min post-ablation [16] and that the maximum lesion SI occurred at approximately 12 min post-ablation in the right ventricle (RV) [4]. In the current study, elevated T_2_ in the maximum T_2_-derived edema ROIs reached a maximum at approximately 60 min post-ablation, possibly arising from a greater capacity for edema development in the LV (compared to thin-walled RV and atria), or the approach used to delineate the edema.

### Study limitations & future work

The earliest T_2_ map acquisition in this study was 3 min after the start of RF energy delivery, and delays typically included catheter withdrawal and waiting for any ablation-induced arrhythmia to settle. T_1_-weighted image acquisition was performed at 13 min at the earliest; therefore any earlier changes among T_1_-derived lesions were missed. Nevertheless, these lesions appeared consistently smaller than the T_2_-derived edema, and were stable or shrinking instead of expanding for time points beyond 13 min post-ablation.

The 2D imaging sequences used in this study were appropriate to maintain relatively short scan times for concurrent imaging of the T_1_-derived lesion and T_2_-derived edema. We anticipate the use of 3D MCLE, a novel sequence using compressed sensing to acquire images of isotropic resolution in a single breath-hold developed for infarct characterization, in future procedures for rapid lesion assessment [33]. Moving towards a future clinical workflow, high-resolution imaging would likely be necessary for pre-ablation targeting--the importance of fine detail to describe complex re-entry circuits is well established in both interventional EP and imaging communities [34, 35]--as well as benefiting lesion assessment, as seen recently [13].

The pro-arrhythmic potential of tissue containing mixed viable and non-viable myocytes and potentially microvascular injury near the periphery of RF lesions was not investigated. However, prior work showed chronic lesions resembling well-circumscribed scar [6], a pattern associated with lower arrhythmogenic potential than heterogeneous scar. The potential for pro-arrhythmia is likely greater in healthy tissue, as was the case in the healthy swine model used for this study. Practically, in the presence of infarcted tissue (as is typical for VT in structural heart disease), lesion pro-arrhythmia may have a small effect since areas ablated are likely to be already ischemic and surrounded by chronic fibrotic tissue. Investigating the properties of ablations on or adjacent to infarcted tissue would build on promising recent results [13] and is an important direction for future studies. In ongoing CMR-based lesion assessment studies, we are also investigating the effect of ablation on the functional properties of local myocardial tissue [36], extending previous studies of infarcted tissue [37].

### CMR-based intra-procedural lesion assessment

Native-contrast CMR imaging reflects ablation-induced tissue changes and facilitates consistent lesion assessment throughout MR-guided ablation. Considering the complexity of the arrhythmogenic substrate, lesion assessment should be repeatable when convenient for the interventionalist, supporting an iterative treatment approach.

Patients undergoing ablation for structural heart disease often exhibit multiple arrhythmia morphologies, suggestive of multiple re-entry circuits. CMR-based lesion assessment could be performed after delivering several ablations towards blocking a target re-entry circuit. Intermittent imaging exams and analysis would require 10–20 min to perform using the techniques employed in the current study (with segmentation not fully optimized for speed).

One proposed workflow for future clinical cases could involve identifying re-entry circuits in prior CMR-derived maps of scar and EP mapping, then delivering several ablations at a re-entry circuit while relying on EP data. T_1_-weighted CMR could be used to visualize a series of lesions created at the re-entry circuit. We suggest that ablation while incorporating this feedback should aim to produce lesions which appear continuous using T_1_-weighted imaging. Based on these T_1_-weighted images, remaining gaps identified at the re-entry circuit would clearly indicate that further ablation is needed, potentially constituting an additional procedural endpoint. T_2_ mapping could be used to interpret possibly discordant EP measures of procedural success, but should not be used alone for lesion assessment as the T_2_-derived edema likely reflects transient injury not contributing to long-term procedural success.

RF lesions could be visualized with the catheter held in place if lesions extend beyond the minimal artifact at the catheter tip, and others have proposed imaging and hardware based solutions for interventions [38, 39]. Catheter withdrawal before imaging (as in this study) may introduce challenges by causing the interventionalist to lose the catheter’s position at the ablation site such that it would need to be directed back precisely for further ablations.

Conscious patients under light sedation may be able to perform breath-holds during intra-procedural CMR imaging, but for those unable, existing respiratory-navigated or real-time sequences could be adapted for lesion imaging [40]. Rapid intra-procedural registration of lesion images to prior scar maps would be needed to provide useful feedback to the interventionalist.

Finally, contrast-enhanced imaging should be reserved to confirm the pattern of ablation at the true completion of the procedure. An alternative sequence recently proposed visualizes both scar and RF lesions with native T_1_ contrast [13]. For either approach, high-resolution coverage of an extended region of the heart, after ablating multiple re-entry circuits, would require a respiratory-navigated acquisition of about 10–20 min. The timing of this acquisition after contrast-agent injection should also be chosen carefully given known contrast agent kinetics.

### Clinical implications

Comprehensive understanding of acute lesion composition and evolution could be applied to both CMR-guided and non-CMR-guided ablation cases. In general, operators should be cognizant of the dynamic nature of edema, which could cause the acute appearance of a successful ablation procedure. The evolving, broad extent of T_2_-derived edema may mask the smaller T_1_-derived lesions (specifically indicated by the ratio of these volumes) which are thought to reflect true procedural success. T_1_-based imaging could potentially constitute an additional procedural endpoint. CMR-based lesion assessment could make a critical difference in scenarios where re-entry circuits appear to be blocked, for instance with more transmural T_2_-derived edema injuring deep epicardial re-entry circuits not reached by the T_1_-derived lesion. These findings may be used to help explain mechanisms of arrhythmia recurrence after acutely successful appearing procedures.

## Conclusions

Native T_1_- and T_2_-based imaging performed concurrently throughout CMR-guided ablations demonstrated that acute T_1_-derived lesions remained at a stable size while the T_2_-derived edema was dynamic, expanded over time, and consistently extended beyond the T_1_-derived lesion. T_2_ quantification provides an unambiguous measure of edema development and severity near ablation sites. Lesion evolution is important for comparing CMR lesion visualization and EP-based endpoints, data which may be acquired at disparate time points. Integrated information on the T_1_-derived lesion and T_2_-derived edema provides a description of acute lesion composition that could be most useful during ablation procedures.

## Abbreviations

ANOVA: Analysis of variance
bpm: Beats per minute
bSSFP: balanced steady state free precession
CMR: cardiovascular magnetic resonance
DSC: dice similarity coefficient
EGM: electrogram
EP: electrophysiology
FOV: field of view
fps: frames per second
H&E: Hematoxylin and Eosin
IR-SSFP: Inversion-recovery balanced steady-state free precession
LMM: linear mixed model
LV: left ventricle/left ventricular
MCLE: multi-contrast late enhancement
MT: Masson’s Trichrome
ROI: region of interest
RV: right ventricle/right ventricular
SD: standard deviation
SE: standard error
SI: signal intensity
TE: echo time
TI: inversion time
TR: repetition time
VT: ventricular tachycardia
